# The α-Amylase and α-Glucosidase Inhibition Capacity of Grape Pomace: A Review

**DOI:** 10.1007/s11947-022-02895-0

**Published:** 2022-08-30

**Authors:** Miluska Cisneros-Yupanqui, Anna Lante, Dasha Mihaylova, Albert I. Krastanov, Corrado Rizzi

**Affiliations:** 1 Department of Agronomy, Food, Natural Resources, Animals, and Environment -DAFNAE, Università Di Padova, Viale dell’Università, 16, Legnaro, PD 35020 Italy; 2 Department of Biotechnology, University of Food Technologies, 26 Maritza Blvd, 4002 Plovdiv, Bulgaria; 3 Department of Biotechnology, Università Di Verona, Strada Le Grazie 15, 37134 Verona, Italy

**Keywords:** Grape pomace, α-Amylase and α-glucosidase inhibition, Functional ingredients, Phenolic compounds, Fiber, Antidiabetic activity

## Abstract

The concept of functional foods is gaining more importance due to its role in maintaining a healthy status and preventing some metabolic diseases. The control of diabetes, in particular type-2 (T2DM), could be considered a big challenge since it involves other factors such as eating habits. From the pharmacological point of view, inhibiting digestive enzymes, such as α-amylase and α-glucosidase, is one of the mechanisms mainly used by synthetic drugs to control this disease; however, several side effects are described. For that reason, using bioactive compounds may appear as an alternative without presenting the complications synthetic drugs available on the market have. The winemaking industry generates tons of waste annually, and grape pomace (GP) is the most important. GP is recognized for its nutritional value and as a source of bioactive compounds that are helpful for human health. This review highlights the importance of GP as a possible source of α-amylase and α-glucosidase inhibitors. Also, it is emphasized the components involved in this bioactivity and the possible interactions among them. Especially, some phenolic compounds and fiber of GP are the main ones responsible for interfering with the human digestive enzymes. Preliminary studies in vitro confirmed this bioactivity; however, further information is required to allow the specific use of GP as a functional ingredient inside the market of products recommended for people with diabetes.

**Graphical abstract:**

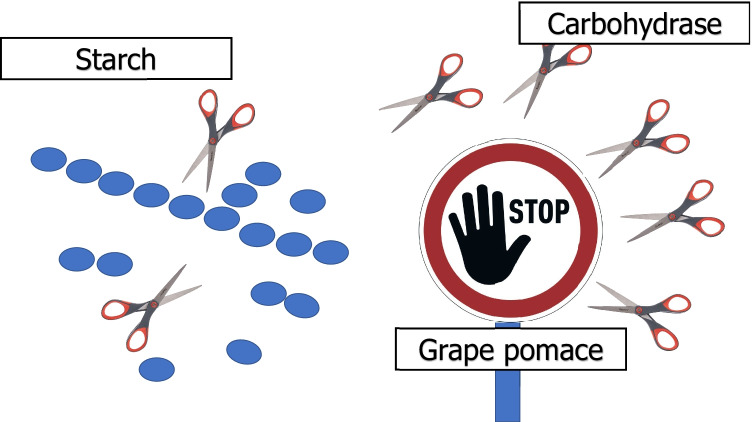

## Introduction

Functional foods contain biologically active compounds, which are responsible for providing health benefits beyond their nutritional capacities (Alongi & Anese, 2021), in particular antioxidant, anti-inflammatory, and antidiabetic activities assessed at in vitro level (Banwo et al., 2021). These capacities turn into health claims after their recognition and authorization, according to the region regulations. For example, according to the European law, it is included inside the Reg. (EU) n. 353/2008 (Alongi & Anese, 2021).

Diabetes is one of the health challenges of the twenty-first century and the number of adults affected by diabetes is more than tripled over the past 20 years. The 10th edition 2021 of the International Diabetes Federation (*IDF*) shows that 537 million adults are currently living with this disease (International Diabetes Federation, 2021). IDF estimates that there will be 643 million adults with diabetes by 2030 and 783 million by 2045. The inhibition of some digestive enzymes, such as α-amylase and α-glucosidase, is one of the options to control this disease by synthetic drugs. However, gastrointestinal discomfort and lactic acidosis are some adverse effects reported (Venkatakrishnan et al., 2019). Currently, there is evidence about the in vitro ability of several fruits, vegetables, and mushrooms to inhibit the activity of these human digestive enzymes (Lin et al., 2022; Papoutsis et al., 2021; Vadivel et al., 2012).

In this regard, Mediterranean diet could be a good option since it is based on local products, mainly of vegetal origin, scarcely processed, and stored for a short time (Sáez-Almendros et al., 2013). However, this food chain generates big amounts of by-products, being necessary to find environmental friendly strategies to revalorize them (Berry, 2019). In this frame, several food industry by-products have been demonstrated to present α-amylase and α-glucosidase inhibition activities, fostering their valorization and the circular economy issue (Fernandes et al., 2020b; Khan et al., 2016; Mahindrakar & Rathod, 2021; Mwakalukwa et al., 2020). For example, the wine industry, which is related to the Mediterranean diet (Ditano-Vázquez et al., 2019), generates more than 9 million tons of grape pomace (GP) per year (Ferri et al., 2017), representing an environmental challenge. An amount of 20–25 kg of GP is estimated to be obtained from 100 kg of grapes and big amounts of this by-product are produced, mainly seasonally (Lavelli, 2021; Muñoz-Bernal et al., 2021).

GP has been recognized to prevent insulin resistance and inflammation (Martínez-Maqueda et al., 2018; Rodriguez Lanzi et al., 2016). In addition, different compounds present in GP such as phenols and fiber were attributed to present antihyperglycemic effects, mainly through the inhibition of the enzymes α-amylase and α-glucosidase (de Paulo Farias et al., 2021; Saikia & Mahanta, 2016). Therefore, the aim of this review was to highlight the potential of GP in inhibiting α-amylase and α-glucosidase enzymes, serving as a possible tool in the diabetes control.

## Methodology

Existing studies related to the GP ability to inhibit α-amylase and α-glucosidase enzymes were gathered to discuss the results currently available. The literature research was carried out in the Scopus database through the period 2002–2022, using initially the keyword “grape pomace.” The search revealed active research on this topic with 1642 articles, out of which 1451, 67, 57, and 49 were research articles, reviews, conference papers, and book chapters, respectively. Most of them (1379) were published from 2012 to 2022, 2021 being the year with the highest number of publications (231).

Then, the research was restricted to scientific papers focused on the inhibition of α-amylase and α-glucosidase by GP, using the keywords “grape pomace + alpha amylase” and “grape pomace + alpha glucosidase.” The number of documents available was reduced to 15 and 20 for the first and second keyword, respectively. This topic has been studied from 2010 onwards, especially in 2020 and 2021. The articles were categorized into the following scientific areas: agriculture and biological sciences (55%), biochemistry (30%), and chemistry (15%). Spain and Chile played the major role in researching this topic. The list of the publications was screened based on the title, authors, and year, and studies not related to the agricultural, biological, and chemistry fields were excluded. After identifying and screening, 10 research articles were selected to discuss the use of GP to inhibit the activity of α-amylase and α-glucosidase.

## Diabetes

Diabetes mellitus (DM) is a chronic non-communicable disease (WHO, 2021) that occurs when the endocrine pancreas is not able to secrete suitable amount of insulin, or when the body does not respond to the insulin it produces. The disease is mainly classified into many types; however, the most common are type 1 (T1) and type 2 (T2) DM. The first one is mainly the consequence of an autoimmune T-cell-mediated reaction against the insulin-producing β-cells of the pancreas. As a result, the body produces very little or no insulin. The second one is the most common type of diabetes, in which hyperglycemia is mainly due to insulin resistance and reduction of insulin production (Gharravi et al., 2018; Mahindrakar & Rathod, 2021; Tan et al., 2019). The insulin resistance is described to be the result of intracellular lipid-induced inhibition of insulin-stimulated insulin-receptor substrate (IRS)-1 tyrosine phosphorylation that determines a reduced IRS-1-associated phosphatidyl inositol 3 kinase activity (Petersen & Shulman, 2006).

A reduced life expectancy is found in both DM types, even if it is shorter in the T1DM compared to T2DM, as a consequence of a higher incidence of cardiovascular diseases and acute metabolic disorders in the former (Wise, 2016). In all forms of diabetes, an early stage diagnosis and management are important to prevent or slow down the potential complications such as diabetic nephropathy, retinopathy, cardiovascular diseases, and diabetic foot ulcer (Khalil, 2017). The potential risk factors, especially for the T2DM, include obesity and unhealthy diets, mainly due to the excessive increase of carbohydrates and fat intake, as well as physical inactivity (Tan & Chang, 2017). Currently, the westernized diet increases the prevalence of specific forms of malnutrition (overweight, obesity, metabolic syndrome, among others), which is exacerbated by the present COVID-19 pandemic (FAO, 2020). Moreover, diabetes is an important risk factor for COVID-19 complications (McGurnaghan et al., 2021; Nassar et al., 2021). Under this point of view, the increasing prevalence of T2DM worldwide is a consequence of a complex interplay of socioeconomic, demographic, environmental, and genetic factors (Tan et al., 2019).

In order to control T2DM, it is encouraged to correct the lifestyle, to reduce the body mass index, and the use of oral antidiabetic drugs. For example, the most used in T2DM management are insulin secretagogues, drugs that reduce insulin resistance, and carbohydrate digestive enzyme inhibitors (AGIs) (Campbell, 2007; Fernandes et al., 2020b). The enzymes α-amylase and α-glucosidase are the main ones inhibited. Both are hydrolases, the activity of α-amylase being to catalyze the starch hydrolysis and it needs the presence of calcium as a metal co-factor. This enzyme is produced in the salivary glands and pancreas, and then it is secreted into the mouth and the small intestine, respectively (Papoutsis et al., 2021). The di- and oligosaccharides obtained after the α-amylase activity undergo further hydrolysis to glucose, carried out by α-glucosidases, located in the brush border of the small intestine (Li et al., 2022).

These enzymes are recognized as targets for modulating the postprandial hyperglycemia (Yang & Kong, 2016), maintaining the overall body glucose levels (Gummidi et al., 2021), and they are present in several plant species due to their bioactive compounds (de Sales et al., 2012).

Acarbose is an AGI, specifically a pseudo-tetrasaccharide that has a nitrogen between the first and second glucose molecules, possessing a particular high affinity for the α-glucosidase enzyme (Tuyen et al., 2021). Both enzymes are inhibited in a competitive way, reducing their affinity to the oligosaccharides from dietary starch as well as decreasing the monosaccharide formation rate (Rosak & Mertes, 2012).

Nevertheless, the carbohydrate digestive enzyme inhibitors are not free from side effects, such as flatulence and diarrhea, abdominal pain, and a reduced nutrient absorption (Wang et al., 2020). In particular, acarbose often generates side effects as a consequence of its non-specific inhibition of α-amylase. This results in an excessive accumulation of undigested carbohydrates in the large intestine (Cardullo et al., 2021).

Taking into account this consideration, the search for more specific and better tolerated α-glucosidase and α-amylase inhibitors with limited effects is an important issue. Therefore, the use of phytochemicals is encouraged, as a consequence of their effectiveness, availability, and low toxicity (de Paulo Farias et al., 2021; Kadouh et al., 2016; Lv et al., 2019). So far, some plant extracts have been reported to counteract T2DM by inhibiting digestive enzymes even stronger than the commercial drugs (Tan & Chang, 2017) or acting synergistically with them (Boath et al., 2012). Natural extracts, especially the ones rich in proanthocyanidins, have shown the ability to inhibit the intestinal α-amylase and α-glucosidase, potentially constituting an alternative to the synthetic AGIs (Yilmazer-Musa et al., 2012).

## Grape Pomace

The wine production represents a huge part of the agriculture and beverage industries. Therefore, it generates a high amount of waste, GP being the most important one (Ilyas et al., 2021). In this regard, 1 kg of GP is produced from each 6 L of wine (García-Lomillo & González-SanJosé, 2017). Among the current applications of this by-product, its uses as fertilizers (especially grape stems), heat producers, and cattle feed are the most highlighted (Antonić et al., 2020; Maragkoudakis et al., 2013; Ribeiro et al., 2015). In addition, GP can be used to produce some value-added components such as edible acids (citric, tartaric, and malic acids) and dietary fiber, as well as ethanol (Ilyas et al., 2021). Moreover, GP is the starting point for preparing alcoholic spirits like Italian grappa (Cisneros-Yupanqui et al., 2021).

After the winemaking process, part of the bioactive compounds in grapes is transferred to the wine; however, a high concentration remains in the residues (Fontana et al., 2013; Gonçalves et al., 2017; José Jara-Palacios et al., 2014; Messina et al., 2019; Ribeiro et al., 2015). Therefore, the recognition of GP as a source of health-promoting components has highly encouraged its use as a food ingredient within the industry (Carmona-Jiménez et al., 2018; Pérez-Jiménez et al., 2009; Rodríguez-Morgado et al., 2015). Among the components found in GP, phenolic compounds and dietary fibers are the most reported in the literature, whose proportion after the winemaking process is up to 85 and 70%, respectively (Rocchetti et al., 2021).

### Grape Pomace Health-promoting Components

Phenolic compounds are found in most plants and more than 10,000 structures have been detected so far (Alqahtani et al., 2013). Their great potentials as powerful bioactive compounds, health-promoting, and disease-preventing have increased the interest in these secondary metabolites in recent years (Ebrahimi & Lante, 2021; Tan & Chang, 2017). The content of phenolic compounds and their composition rely on the growth region, climate, and grape variety, among other factors related to the winemaking process (Muñoz-Bernal et al., 2021). The phenolic compounds in grape berries are distributed in the pulp, seeds, and skin, these two last ones being the main sources (Gonçalves et al., 2017), especially of procyanidins (Álvarez et al., 2012). Part of these bioactive compounds remain in the GP after the winemaking, along with important quantities of catechins, epicatechins, and flavan-3-ols, mainly due to the hydrogen bonds and their hydrophobicity (Barba et al., 2015; Cisneros-Yupanqui et al., 2020a; Muñoz-Bernal et al., 2021). In addition, the phenolic compounds present in GP have shown a good stability, especially as a powder, during the storage (Cisneros-Yupanqui et al., 2020b), showing its potential to be considered as a food ingredient. So far, the phenolic compounds present in GP have had different applications, as summarized in Table 1. In all the cases, the concentration of phenolic compounds and antioxidant activity has increased after the fortification with GP, regardless the food matrix (Fernández-Fernández et al., 2022; Lavelli et al., 2016; Rainero et al., 2021) and the type of GP employed. In some cases, the addition of GP was useful to delay the lipid oxidation (Cisneros-Yupanqui et al., 2020a; García-Lomillo et al., 2017) not only the one derived from the winemaking process, but also the GP from the juice industry, when applying it in frozen salmon burgers at 2% (Cilli et al., 2019). Moreover, the addition of GP has increased the characteristics of a fortified wheat bread and pasta, presenting a better volume, firmness, taste intensity, and color (Šporin et al., 2018; Tolve et al., 2020). However, the firmness and consistency of a GP-fortified yogurt did not change considerably when comparing to the control (Iriondo-DeHond et al., 2020).

**Table 1:** Phenolic compounds from grape pomace used in the fortification of different food products

Winery by-product	Food matrix	Grape by-product concentration	Results	References
Red (Valpolicella) grape pomace (GP) from winemaking	Corn oil	1, 2, 3% of GP powder	-Epicatechins were the most predominant phenolic compounds found in GP -Corn oil + 1% GP delayed the corn oil oxidation by 10%	Cisneros-Yupanqui et al. (2020a)
GP, seeds, and skin from winemaking	Home-made yogurt	5 mg/mL of winery by-products	-GP, seeds and skin fortified yogurts obtained higher TPC and antioxidant activity than the control -The total lactose and fat percentage were lower in yogurts supplemented with GP, seeds, and skin -The firmness and consistency of the yogurt did not change significantly when adding GP, seeds, and skin along 21 days of storage	Iriondo-DeHond et al. (2020)
Unfermented white GP with a further selection of skins	-Tomato puree -Flat bread	3% and 10% of grape skin for tomato and bread, respectively	-Almost all the white grape skin phenolics were found in the enriched foods -Proanthocyanidin solubility was lower in bread than in tomato puree	Lavelli et al. (2016)
Red (Merlon) and white (Zelen) GP from winemaking	-Wheat bread	6%, 10% and 15% of GP	-The TPC was improved mostly in the bread fortified with 15% of GP flour, Merlot having the highest concentration -The TPC and the antioxidant activity were highly correlated with the GP flour addition -GP flour addition has an influence on the bread volume, firmness, taste intensity, and crumb and crust color	Šporin et al. (2018)
GP, seeds and skin from red winemaking	-Frozen beef patties	2% of GP, seeds, and skin	-The frozen beef patties fortified with grape skins were the most effective in inhibiting TBARS, due to the TPC	García-Lomillo et al. (2017)
Red (Corvina) GP with a further selection of skins	-Durum wheat semolina	5 and 10% of GP, replacing semolina	-The TPC and antioxidant activity of the fortified pasta was enhanced -The fortified pasta obtained a higher fiber content, from 5.6 to 8.2% than the control (3%)	Tolve et al. (2020)
Red (Cabernet) GP with a further selection of skins	-Breadsticks	5 and 10% of GP, replacing the common wheat flour	-The TPC and antioxidant activity of the fortified breadsticks were enhanced, 10% addition obtaining significantly the highest values	Rainero et al. (2021)

On the other hand, fiber, especially the dietary one, has been studied to promote diverse beneficial effects such as improving the gastrointestinal function, reducing the low-density lipoprotein (LDL) cholesterol, and moderating the response of the postprandial insulin response (Mildner-Szkudlarz et al., 2013). In addition, fiber helps in reducing the risk of cardiovascular diseases and it is defined as an edible carbohydrate analogous, digestion and absorption resistant through small intestinal tract with a fermentation (partial or complete), in the large intestine (Solari-Godiño et al., 2017). Dietary fiber can be classified as soluble and insoluble, the former including β-glucans, hemicellulose, pectin, and oligosaccharides (Dong et al., 2022). The soluble dietary fiber is recognized for lowering glucose levels and controlling obesity in patients with T2DM (Xie et al., 2021), while insoluble fiber prevents constipation and hemorrhoids by going fast through the gastrointestinal tract, providing bulk to the feces (Ain et al., 2019).

GP has been reported to be a rich source of fiber (from 44.2 to 62.6%), which allows its use into bakery and dairy products (Fernández-Fernández et al., 2019, 2022; Oladiran & Emmambux, 2018; Rainero et al., 2021). Furthermore, grape by-products contain mainly cellulose, hemicelluloses, glycans, and pectin (Fontana et al., 2013; Mildner-Szkudlarz et al., 2013; Oladiran & Emmambux, 2018), and the insoluble dietary fraction, such as lignin, has been the most reported one in this type of residue, presenting good water and oil holding capacity as well as antioxidant activity (Mildner-Szkudlarz et al., 2013; Saikia & Mahanta, 2016). The term antioxidant dietary fiber has been introduced to define a products that present both natural antioxidants and the beneficial effects of dietary fiber (Sánchez-Alonso et al., 2007). For example, it could present antioxidant properties and inhibit lipid and protein oxidation (Garcia-Lomillo et al., 2016; Lavelli, 2021; Marchiani et al., 2016; Sáyago-Ayerdi et al., 2009). The association and health effect of dietary fiber and phenolic compounds are appreciated at the large intestine level (Solari-Godiño et al., 2017). Moreover, the ability of phenolic compounds to modify the gut microbiota, improving and inhibiting the growth of beneficial and pathogenic bacteria, respectively, was reported (Gowd et al., 2019).

## Grape Pomace as α-Amylase and α-Glucosidase Inhibitors

Phenolic compounds have been recognized for presenting several bioactivities, including the antidiabetic one, which is mostly related to their capacity of decreasing the postprandial glycemic levels, especially through the inhibition of human digestive enzymes (Alqahtani et al., 2013; Martinez-Gonzalez et al., 2017; Tan & Chang, 2017), with a consequent reduced dietary starch digestion and absorption (Hogan et al., 2011). The inhibition of these enzymes by diverse type of phenolic compounds has been well studied in the literature (Oladiran & Emmambux, 2018; Rocha et al., 2020; Shobana et al., 2009). Phenols are the most involved in these bioactivities (Kato-Schwartz et al., 2020) by binding to either the sites or the substrate of the digestive enzymes, making them inactive (Oladiran & Emmambux, 2018). Some characteristics of phenolic compounds such as the molecular weight, number, and position of substitution are suitable for their digestive enzyme inhibitory activity (Fernandes et al., 2020b). In addition, flavonoids have been recognized to interfere with the α-amylase activity by forming covalent bonds with starch during cooking and in the stomach, decreasing its availability as a substrate for the enzyme (Takahama & Hirota, 2018). Procyanidins of grape seeds are responsible for presenting health-promoting effects such as antioxidant and antihyperglycemic by inhibiting α-amylase and α-glucosidase enzymes (Fernandes et al., 2020a; Takahama & Hirota, 2018; Yilmazer-Musa et al., 2012). These compounds are polymers of flavan-3-ols, which are formed exclusively by catechin and/or epicatechin units (Álvarez et al., 2012). Procyanidins have more potential interaction sites than the monomeric phenolic compounds, so they could crosslink easily with different molecules, such as enzymes (Lavelli et al., 2016). On the other hand, proanthocyanidins have been shown to inhibit these key enzymes, due to their high polymerization degree and numerous hydroxyl groups (Huamán-Castilla et al., 2021). In particular, the high degree of polymerization of these molecules present in ripe fruits showed more potent inhibition of α-amylase and α-glucosidase than the less-polymerized ones, which are typically present in unripe fruits (Zhang et al., 2020).

In addition, proanthocyanidins and anthocyanins have been demonstrated to exert a major role in inhibiting α-amylase and α-glucosidase, respectively (Lavelli et al., 2015), in comparison to acarbose (Yilmazer-Musa et al., 2012). Regarding catechins, it was suggested that galloylated catechins and catechol-type catechins present a higher α-amylase inhibitory activity than non-galloylated and pyrogallol-type ones (Takahama & Hirota, 2018; Yilmazer-Musa et al., 2012). Moreover, galloyl groups from catechins were related to inhibiting the α-amylase activity by binding other sites than the active one as well as presenting good affinity to human α-amylase (Miao et al., 2014). Catechins were also found to suppress and enhance the amylopectin and amylose digestion, respectively, by forming starch-catechins complexes without modifying the α-amylase activity (Liu et al., 2011). In addition, resveratrol could delay the activity of both enzymes (Fernandes et al., 2020a). However, phenolic compounds enhance or decrease the α-amylase activity when low and high concentrations are used, respectively (Yang & Kong, 2016). In general, tannins have been reported to inhibit α-amylase, while α-glucosidase is inactivated by smaller phenolic compounds such as phenolic acids (Barrett et al., 2013; Oladiran & Emmambux, 2018). Besides the potential of phenolic compounds in this bioactivity, several factors such as the concentration in food, bioaccessibility, absorption, metabolism, and bioavailability can maximize the antidiabetic capacity of this compounds (Chen et al., 2019; de Paulo Farias et al., 2021). For example, factors such as pH and temperature may modify the interaction between the phenolic compounds and proteins (including the digestive enzymes), as reported by Martinez-Gonzales et al. (2017).

On the other hand, the molecular interactions mostly recognized between the enzymes and phenolic compounds are van der Waals, electrostatic forces, and hydrogen as well as hydrophobic binding (Martinez-Gonzalez et al., 2017), which have been related to inhibit the enzymes in a non-competitive way (Rocha et al., 2020; Yang & Kong, 2016). Therefore, the inhibitor can bind to either the free enzyme or the complex enzyme–substrate (Rocha et al., 2020). In addition, this kind of inhibition has been previously found in GP (Oladiran & Emmambux, 2018). Some phenolic compound inhibition has been observed to be in a competitive way, especially the one from quercetin and caffeoylquinic acid (Martinez-Gonzalez et al., 2017). However, non-covalent interactions are recognized to be the key of the enzymatic inhibition since they represent the basis of reversible inhibitions, which may be useful within some medical treatments (Martinez-Gonzalez et al., 2017). The number of galloyl ester groups and the polymerization degree are the main characteristics of phenolic compound structure that have an influence on their interactions with proteins (Lavelli et al., 2016).

Additionally, the α-amylase activity has been related to the insoluble fiber content and to the limited enzyme accessibility to the substrate, due to network of starch and enzyme by the fiber (Saikia & Mahanta, 2016). Moreover, insoluble dietary fiber has a higher inhibitory effect on α-glucosidase than on α-amylase, and this activity may be related to the inhibitors present on the surface of the fiber as well as the trapping capacity of the porous fiber network (Yang et al., 2019). However, the soluble dietary fiber is the most associated with the postprandial glucose response by reducing the glucose absorption (Oladiran & Emmambux, 2018).

GP has been identified as an α-glucosidase and α-amylase inhibitor (Table 2), showing, especially the red varieties, a possible potential in the management of diabetes (Fernandes et al., 2020a; Hogan et al., 2010; Kadouh et al., 2016; Kato-Schwartz et al., 2020). Table 2 shows that yeast α-glucosidases are usually employed in research (Kong et al., 2019); however, the mammalian enzymes are more biologically relevant since they are more comparable to those acting in the human intestinal tract (Kadouh et al., 2016). In addition, GP has lowered the starch digestibility rate and the estimated glycemic index (Oladiran & Emmambux, 2018; Rocchetti et al., 2021; Tolve et al., 2020). Moreover, GP was employed to fortify yogurt, showing a higher α-glucosidase inhibition activity (Fernández-Fernández et al., 2022; Iriondo-DeHond et al., 2020). Seeds present in GP powder as well as their extract have been described to inhibit α-amylase and α-glucosidase, respectively, the efficiency being comparable and higher than acarbose (Yilmazer-Musa et al., 2012). This activity was even more potent than the one exerted by isolated catechins in the case of α-amylase, while epigallocatechin gallate (EGCG) has reached a more significant effect on α-glucosidase inhibition (Yilmazer-Musa et al., 2012). On the other hand, the inhibition of α-glucosidase has shown to reduce the postprandial hyperglycemia in diabetic mice when they were fed with grape skins (Hogan et al., 2011), while a recent study has showed GP does not have an effect on glucose absorption, but inhibiting the amylase activity (Kato-Schwartz et al., 2020).

**Table 2:** Grape pomace as α-glucosidase and α-amylase inhibitor

References	Winery by-product	Stabilization protocol	Extraction method	Enzyme inhibition activity	Methods of study	Results
Fernandes et al., (2020a)	Red (Syrah-Seibel), white (Muscat), and mixed grape pomaces from winemaking	Seeds and skin were milled and sieved (10-mesh sieve). Then, they were stored at − 80 °C	Preparation of a 1:10 methanolic extract with a further shaking, ultrasonic bath, filtration, and concentration	α-Amylase from porcine pancreas	In vitro	-Red GP obtained the highest TPC and AOX activity than white and mixed pomace -The α-amylase inhibition percentage of red GP was the highest reported (almost 94%) while the one from mixed GP was the lowest (72.69%) at 10 mg/mL of phenolic concentration -Catechin and procyanidin B2 were the most predominant phenolic compounds, maybe responsible for the α-amylase inhibition activity
Fernández-Fernández et al. (2019)	Red (Tannat) grape pomace from winemaking	Seeds and skin were separated manually. Then, the skin was dried at 40 °C up to constant weight (24 h) with a further milling	Preparation of different extracts with ethanol at 95%, methanolic and formic acid with a further ultrasonic extraction, filtration, concentration, and lyophilization	Commercial α-glucosidase from *Saccharomyces cerevisiae* type I	In vitro	-Almost 50% of fiber content -The hydro alcoholic acid extraction obtained higher total phenolic content than the methanolic and aqueous ones -The hydro alcoholic acid extract obtained the best α-glucosidase inhibition capacity (IC_50_ 889 µg/mL) with almost 90% inhibition percentage at 10 mg/mL of phenolic concentration
Hogan et al. (2010)	Red (Cabernet Franc) and white (Chardonnay) grape pomaces from winemaking	The samples were immediately freeze-dried upon receiving and then ground	The extraction was carried out with ethanol 80% (1:10 ratio) with a further shaking, filtration, and evaporation of the solvent	α-Glucosidase from yeast and rat intestine	In vitro	-Both GP extracts inhibited the intestinal α-glucosidase -Red GP inhibited both α-amylases activity in a higher amount than white GP (almost 60 and 47% in the case of α-glucosidase from yeast and rat intestine, respectively) at 1.5 mg/mL of phenolic concentration
Kato-Schwartz et al. (2020)	Red (Merlot) grape pomace from winemaking	The pomace was dried in a convection oven at 80 °C for 36 h with a further milling	Preparation of different extracts (40% ethanol and 60% distilled water) with a 1:50 (m/v) ratio, with a further shaking, centrifugation concentration of the solvent, and lyophilization	Pancreatic and salivary α-amylase, intestinal α-glucosidase	Study in vitro*, *in silico*,* and in vivo	-The most abundant phenolic compounds found were epicatechin, catechin, quercetin, myricetin, isorhamnetin glycoside derivatives, malvidin-3-O-glucoside, and peonidin-3-O-glucoside -Salivary α-amylase inhibition was stronger than pancreatic amylase (IC_50_ values 90 and 143 µg/mL, respectively) -No inhibition of α-glucosidase was observed
Kong et al. (2019)	Red (Cabernet Sauvignon) grape seeds from winemaking	The extract from the grape seeds was provided from the winery. The formation of inclusion complexes with sulfobutyl ether-β-cyclodextrin was performed in a ratio 1:10 (grape seed extract:sulfobutyl ether-β-cyclodextrin)	ND	α-Glucosidase and α-amylase from baker’s yeast	In vitro	-At concentration higher than 2 mg/mL, the inclusion complex inhibited α-glucosidase activity stronger than acarbose with an IC_50_ of 1.188 and 1.035 mg/mL, respectively -At the same conditions, the IC_50_ for inhibiting amylase activity was 0.513 and 0.587 mg/mL for the inclusion complex and acarbose, respectively
Kadouh et al. (2016)	Red (Chambourcin, Merlot, Norton, Petit Verdot, Syrah and Tinta Cão) grape pomaces from winemaking	The pomaces were immediately dried in a food dehydrator at 35 °C for 28 h and then separated from stems to be ground	The extraction with aqueous acetone (50%) at 0.1 g/mL (GP powder/solvent) was carried out with a further filtration, concentration, and lyophilization	α-Glucosidase from rat intestine	In vitro	-Merlot GP obtained the highest TPC (0.29 mg/mL) while Petit Verdot the lowest (0.06 mg/mL) -Tinta Cão GP presented the highest concentration of malvidin chloride, delphinidin chloride, epicatechin gallate, and resveratrol, if compared with the rest of the varieties studied -Tinta Cão grape pomace obtained the strongest α-glucosidase inhibition while Petit Verdot reached 7%, both at 0.5 mg/mL of dry extract
Lavelli et al. (2016)	White grape pomace from winemaking	The GP was sieved (5 mm) to separate the skins from the seeds. The skins were frozen and then dried at 55 °C for 48 h (*a*_w_ < 0.3). Then, it was ground and sieved	1 g of grape skin was extracted with 20 mL of methanol/water/formic acid (70:29.9:0.1, v/v/v) for 2 h at 60 °C with continuous stirring. Then, a centrifugation (10000* g* for 10 min) and a re-extraction was carried out twice	α-Glucosidase and α-amylase from intestine and pancreas, respectively	Study in model foods: tomato puree and bread	-Quercetin and kaempferol derivatives were identified in grape skin -Enzyme inhibition by the enriched foods was higher than their respective controls
Lavelli et al. (2015)	Red (Barbera, Dolcetto, and Albarossa) and white (Chardonnay, Muller Thurgau, Cabernet Sauvignon, and Moscato Bianco) grape pomace from winemaking	The stalks were separated from the pomaces and then sieved (5 mm) to obtain the seeds. The seeds were dried at 55 °C for 48 h for a further grinding and then defatted by SC-CO_2_ at a pressure of 500 bar at 50 °C	1 g of grape seeds was extracted with 16 mL of methanol/0.1% HCl for 2 h at room temperature with continuous stirring. Then, a centrifugation was carried out at 10000* g* for 10 min. A re-extraction was performed twice with 12 mL of the same solvent and then the 3 supernatants were collected	α-Glucosidase and α-amylase from intestine and pancreas, respectively	In vitro	-The major phenolic compounds found were proanthocyanidins -The main factor for the α-glucosidase inhibition was the grape variety, Albarossa and Barbera obtaining the weakest inhibitory properties -Good correlations were found between the content of phenolic/proanthocyanidin contents and the inhibition of α-amylase
Huamán-Castilla et al. (2021)	Red (Carménère) grape pomace from winemaking	The GP (skin and seeds) was reduced down (2 mm) using a blender	5 g of GP was mixed with 110 g quartz sand. The mixture was placed in an hot pressurized liquid extraction (HPLE) device, using pure water, water-glycerol (15%), and water–ethanol (15%) at 90, 120 and 150 °C, applying 10 MPa, one extraction cycle, 150% washing volume, 250 s nitrogen purge time, and 5 min static extraction. The final matrix/solvent ratio was 1:10	α-Glucosidase from *S. cerevisiae* and porcine pancreatic α-amylase	In vitro	-The higher the HPLE temperature, the higher the TPC, reaching 143% when using pure water as solvent -The highest antioxidant activity was found when using water-glycerol as solvent (27% more than the control) -1000 µg/mL of the water–ethanol extract at 90 °C decreased the activity of α-amylase and α-glucosidase by 56 and 98%, respectively -Acarbose inhibited the activity of both enzymes by 56 and 73% for α-amylase and α-glucosidase, respectively, at the same concentration (1000 µg/mL)

Another factor to consider when assessing the GP inhibitory activity is the type of study. After the preliminary in vitro screening, it is necessary to carry out an in vivo model to understand some factors such as the bioavailability and the physiological response to the GP components (Alongi & Anese, 2021; Gerardi et al., 2020; Kato-Schwartz et al., 2020). However, human clinical trials are mandatory required (Reg. (EU) n. 353/2008) for obtaining a health claim (Alongi & Anese, 2021).

## Conclusion

The present review has highlighted the importance of GP as a promising α-amylase and α-glucosidase inhibitor, due to the complexity of its components. Diverse phenolic compounds and fiber are the constituents more related to this bioactivity, beyond their traditional properties. In addition, the GP inhibition of α-amylase and α-glucosidase has been showed to remain also in the fortified food products with this ingredient. However, it is crucial to focus on the kind of study performed since the majority is preliminary at an in vitro level, clinical trials being necessary to reach stronger conclusions. Although the studies reported in this review were carried out in the GP extract, the use of the whole GP would be more convenient because it is easier to use and eco-friendly, and all the bioactive compounds involved in the α-amylase and α-glucosidase inhibition activity may remain. The GP capacity of inhibiting α-amylase and α-glucosidase along the time is another factor to take into consideration since several reactions between the internal GP components can take place during its storage, modifying its bioactivity. This review deals with the GP obtained after the winemaking process; however, scarce information is available regarding the utilization of the exhausted GP recovered after the production of distilled spirits, whose bioactivity was barely pointed out. The valorization of these by-products as functional ingredients within the food industry as α-amylase and α-glucosidase inhibitors could encourage the circular economy approach of a more sustainable production.
